# Supplementary material for: Insecticide resistance status of indoor and outdoor resting malaria vectors in a highland and lowland site in Western Kenya
Source: PLoS One. 2021 Mar 1;16(3):e0240771. doi: 10.1371/journal.pone.0240771 (PMC7920366; doi:10.1371/journal.pone.0240771)
Supplement: Attachment — Submitted filename: comments.docx [file pone.0240771.s001.docx]

**Comments**:

This study intends to show the insecticides resistance between indoors and outdoors resting vectors in highlands and lowlands

This study has a major drawback in methodology.

To start with the ecological behavior of An. gambiae s.l is to feed indoor and rest indoor for An. gambiae s.s. while for An. arabienses is feeding indoor and resting outdoor or feed outdoor and rest outdoors. Due to the wide coverage of the insecticide treated surfaces (LLINs and RS), mosquitoes have changed the feeding and resting behavior to forfeit the effect of the insecticides treated surfaces. Due to this, higher proportions have found to feed and rest outdoors regardless of the vector species.

Sampling:

The samplings of vectors were done indoor and outdoor. The vector after feeding indoor they get out to rest and search for oviposition site. This is indeed a challenge to mark that this mosquito was found resting outdoor while it fed indoor. The physiological and enzymatic dynamics in mosquitoes is mostly reflected by blood meal source. There is no barrier between indoor and outdoor resting populations hence free mating is possible. In first place, authors should be aware that resistance is genetical and is clearly related age of the mosquito. These mosquitoes share breeding sited and feeding sources. The emerging young mosquitoes resting outdoor before searching for host. The outdoor collection is probably that it collected emerged and gravid mosquitoes searching for oviposition site. The methods were weak not to show the separation of population fed outdoor or indoor. So collection alone cannot provide a guarantee of the biochemical processes to be elevated by resting position.

I personally reject this paper as the basis used of outdoor and indoor resting have no barrier separation between the populations collected. Since resistance is genetical and biochemical process is influenced by blood meal sources. No evidence of where the mosquitoes fed (i.e. blood meal source). That was not shown so as to give the=at strong evidence.

This study can compare the enzyme changes and resistance and enzyme presentation between lowland and highland but not between indoor and outdoor resting mosquitoes

More specific comments are uploaded in attached document.
