# Supplementary material for: Insecticide resistance status of indoor and outdoor resting malaria vectors in a highland and lowland site in Western Kenya
Source: PLoS One. 2021 Mar 1;16(3):e0240771. doi: 10.1371/journal.pone.0240771 (PMC7920366; doi:10.1371/journal.pone.0240771)
Supplement: Attachment — Submitted filename: Response to reviewers.docx [file pone.0240771.s002.docx]

**Response to reviewer comments**

The following are our point-by-point responses to the comments raised by the reviewers. We really thank the reviewers for their constructive criticism and insights that have helped to improve this paper.

**Reviewer Comments:**

**Reviewer #1**

**Reviewer:** Line 73 to 75 « Despite the observed improvement in malaria incidence and prevalence in many parts of sub-Saharan Africa, transmission is increasing in several countries [2,3]. » I don’t think the term « improvement » is appropriate and the sentence doesn’t make sense

**Response:** The sentence has been revised and now reads” Despite the observed achievements in malaria reduction, many parts of sub-Saharan Africa still suffer greatly from the disease”….**Line 72-74**

**Reviewer:** Line 80 to 81 « Insecticide resistance in malaria mosquitoes is linked to presence and increase in metabolic detoxification enzymes, target site insensitivity and behavioural resistance [11]. » the sentence is not clear can the author rephrase the sentence to improve understanding?

**Response:** The sentence has been revised to read “Insecticide resistance in malaria mosquitoes has been linked to target-site insensitivity, elevated levels of metabolic detoxifying enzymes”……**Line 77-78**

**Reviewer:** Line 84 to 86 « Detoxification enzyme systems that have been reported to confer resistance include three major families of enzymes; the cytochrome P450monooxygenases, β-esterases, and the Glutathione S-transferases. » please write esterase as you are refering to enzymes families

**Response:** This has been addressed …**Line 82**.

**Reviewer:** Line 92 to 95 « The increasing levels of insecticide resistance in malaria mosquitoes have been associated with continuous exposure to insecticides in LongLasting Insecticide Nets( LLINs) [23,24] and agro-chemicals such as pesticides due to the creation of selection pressures [25-27]. » The phrase is not clear and need to be rewritten to improve understanding

**Response:** The statement has been rewritten and now it reads **“** The increasing levels of insecticide resistance in malaria mosquitoes is believed to be mainly caused by scaling up of insecticidal treated nets (ITNs) [23,24] and indiscriminate use of agro-chemicals for controlling crop pests in agriculture[25-27] ..**Line 88-90**

**Reviewer:** The introduction is not straight forward and is poorly written the authors need to revise this section carefully to improve understanding

**Response:** The introduction section has been revised to improve understanding.

**Reviewer:** Line 120 « The study was carried out in the lowland site of Kisian (0.0749° S, 34.6663° E, 1,137m) in Kisumu county … » The geographical coordinates is not appropriate can the authors check for the correct coordinates ?

**Response**: The geographical coordinates provided for Kisian are the correct one “00.0749° S, 034.6663° E, altitude 1,137-1,330 m above sea level)”

**Reviewer:** Line 127 to 128 « There is extensive use of agrochemicals on these farms which could have a potential role in the mediation of resistance to insecticides. » Are anopheline breeding habitats found in tobacco farms and what type of insecticides are used in these farms please provide more information. Please also say how frequent insecticides are sprayed in these farms. How distant are these farms from the village.

**Response:** The sentence has been modified from line 120 -125 and also cited Wanjala et al 2015 which has detailed information on the agrochemicals used in the study area.

**Reviewer:** Mosquitoes were collected in how many houses per site and outdoor places please include information.

**Response**: The information on the number of houses sampled and outdoor places has been included under mosquito sampling section …. ‘Thirty (30) houses were randomly selected per site and resting mosquitoes collected from 06:00 to 09:00 h……” **Line 130-139**

**Reviewer:** Line 156 to 160 « First filial generation (F1) females raised from field-collected adults that were resting either indoors or outdoors, that were 3-5 -day old, were tested for susceptibility using the standard WHO tube bioassays (WHO, 2016) against discriminating doses of five insecticides selected from three classes: (i)Pyrethroids - (0.05% deltamethrin, 0.75% permethrin and (0.05% Alphacypermethrin); and (ii) organophosphate - (5% malathion). » The author say they tested 5 insecticides only a list of four insecticide is provided please correct.

**Response**: This has been corrected in the text, four insecticides from 2 classes of insecticides were used **Line161.**

**Reviewer:** Line 166 to 167 « Mortality was defined as the inability of the mosquitoes to stand or to fly in a coordinated manner. » is this mortality or knock down effect please correct.

**Response**: The statement has been deleted from the text.

**Reviewer:** Line 175 to 176 « After pre-exposure to PBO, the mosquitoes were immediately exposed to the three pyrethroids (deltamethrin, permethrin and alphacypermethrin) for an additional hour. » Where mosquitoes exposed to all three insecticides at once or seperately to each insecticide please clarify

**Response**: The sentence has been corrected and now reads “After pre-exposure to PBO, the mosquitoes were immediately exposed to each of the three pyrethroids (deltamethrin, permethrin and alphacypermethrin) separately for another hour….**Line 177-178**

**Reviewer:** There are data missen in this part the author need to give the number of gravid and non gravid females collected outdoor and indoor in each site. They could alsoindicate the number collected during each season.

**Response:** We have included the data for the gravid females that were used for the study from line 155-157.

**Reviewer:** The number of mosquitoes tested for each insecticide need to be provided

**Responses**: The number of mosquitoes tested for each insecticide was 150 according to the WHO 2016 Insecticide resistance testing procedures the information is already provide in the text.. **Line 163**

**Reviewer:** The authors say that the percentage is in bracket but it is not clear how they did the calculations of the percentage of An. funestus in Kisian 122(19%) in Kimaeti they also wrote 167 (23.16) can they provide explanation for their calculations or correct. The author also need to check their calculations the row on An. arabiensis in Kisian the total in the column and line also need to be corrected.

**Response**: The figures in table 1 has been corrected

**Reviewer #2**

This study intends to show the insecticides resistance between indoors and outdoors resting vectors in highlands and lowlands This study has a major drawback in methodology. To start with the ecological behavior of An. gambiae s.l is to feed indoor and rest indoor for An. gambiae s.s. while for An. arabienses is feeding indoor and resting outdoor or feed outdoor and rest outdoors. Due to the wide coverage of the insecticide treated surfaces (LLINs and RS), mosquitoes have changed the feeding and resting behavior to forfeit the effect of the insecticides treated surfaces. Due to this, higher proportions have found to feed and rest outdoors regardless of the vector species.

Sampling: The samplings of vectors were done indoor and outdoor. The vector after feeding indoor they get out to rest and search for oviposition site. This is indeed achallenge to mark that this mosquito was found resting outdoor while it fed indoor. The physiological and enzymatic dynamics in mosquitoes is mostly reflected byblood meal source. There is no barrier between indoor and outdoor resting populations hence free mating is possible. In first place, authors should be aware thatresistance is genetical and is clearly related age of the mosquito. These mosquitoes share breeding sited and feeding sources. The emerging young mosquitoesresting outdoor before searching for host. The outdoor collection is probably that it collected emerged and gravid mosquitoes searching for oviposition site. Themethods were weak not to show the separation of population fed outdoor or indoor. So collection alone cannot provide a guarantee of the biochemical processes tobe elevated by resting position. I personally reject this paper as the basis used of outdoor and indoor resting have no barrier separation between the populations collected. Since resistance isgenetical and biochemical process is influenced by blood meal sources. No evidence of where the mosquitoes fed (i.e. blood meal source). That was not shown so as to give the=at strong evidence.

This study can compare the enzyme changes and resistance and enzyme presentation between lowland and highland but not between indoor and outdoor resting mosquitoes More specific comments are uploaded in attached document.

**Response:** Our study looked at the differences in insecticide resistance between indoor and outdoor resting mosquitoes. The focus was not on biting mosquitoes as mosquitoes are likely to be influenced by where the host might be, whether indoor or outdoor, to take a blood meal. They are likely to make a choice, after taking a blood meal, between resting indoors or outdoors based on their preferences as exophilic or endophilic vectors. We acknowledge that the taking of a blood meal by mosquitoes might influence their insecticide resistance status through the raising of biochemical enzymes to tolerate insecticides. This made us not to use the female mosquitoes collected but their F1 progeny so that we can understand whether resting behaviour is a consistent behaviour. We have clarified our objective and some parts of the methodology, which should make our methodology clear to answer the points raised by the reviewer 2. We strongly disagree with the reviewer that we should have shown where the mosquitoes took their blood meals. We also reject the suggestion that we “can compare the enzyme changes and resistance and enzyme presentation between lowland and highland”. This has no scientific basis. The methodology in our study was not weak as alleged by the reviewer but probably he may not have clearly understood it.

**Editorial comments**

**To the editor:**

Figure 1 image has been removed from the manuscript.

We have also taken the ethics statement to the methods section
